# Study on the species composition and ecology of anophelines in Addis Zemen, South Gondar, Ethiopia

**DOI:** 10.1186/s13071-018-2701-3

**Published:** 2018-03-27

**Authors:** Mizan Kindu, Esayas Aklilu, Meshesha Balkew, Teshome Gebre-Michael

**Affiliations:** 1 School of Medicine, Mada Walabu University, Bale-Robe, Ethiopia; 2 Department of Biology, Mada Walabu University, Bale-Robe, Ethiopia; 3 Aklilu Lemma Institutes of Pathobiology, Addis Ababa University, Addis Ababa, Ethiopia

**Keywords:** *Anopheles gambiae* (*s.l.*), Sheni stream, Addis Zemen, Ethiopia

## Abstract

**Background:**

Malaria is a public health problem in Ethiopia and its transmission is generally unstable and seasonal. For the selection of the most appropriate vector control measures, knowledge on the ecology of the vector is necessary at a local level. Therefore, the objectives of this study were to document the species composition, breeding habitat characteristics and occurrence of anopheline larva in Sheni stream and the vectorial role of the prevailing *Anopheles* in relation to malaria transmission in Addis Zemen, Ethiopia.

**Methods:**

Immature anophelines were sampled from breeding habitats and characteristics, such as water temperature, turbidity, water current, water pH and other variables, of the habitats were measured from October 2011 to February 2012. Adult anophelines were sampled inside human dwellings using space spray and Center for Disease Control light traps. Artificial pit shelters and clay pots were also used for outdoor adult collections. Anophelines collected were identified using morphological key. The enzyme-linked immunosorbent assay was applied to detect circumsporozoite proteins of *Plasmodium* and source of blood meals.

**Results:**

A total of 6258 *Anopheles* larvae were collected and identified morphologically. Five anopheline species were found: *An. gambiae* (*s.l.*), *An. cinereus*, *An. demeilloni*, *An. christi* and *An. pretoriensis*. *Anopheles gambiae* (*s.l.*) existed in most of the habitats investigated*.* Only the former three species were captured in the adult collections. Sun-lit Sheni stream, rain pools, hoof prints, drainage and irrigation canals were found to be habitats of larvae. *Anopheles gambiae* (*s.l.*) larvae were most abundantly sampled from sand mining and natural sand pools of Sheni stream. Multiple regression analysis showed that clear, permanent and temporary habitats devoid of mats of algae were the best predictors of *An. gambiae* (*s.l.*) larval abundance. It is also the responsible malaria vector in the study area and exhibits anthropophilic and endophagic behaviour.

**Conclusions:**

The malaria vector *An. gambiae* (*s.l.*) was found in Addis Zemen throughout the study period from both adult and larval collections. Sheni stream is the main larval habitat responsible for the occurrence of anopheline larvae during the dry season of the study area when other breeding sites perish.

## Background

Malaria is one of the main public health problems globally and is endemic in 91 countries of the world. Its incidence is estimated to be 212 million cases in 2015; of these, 90% of the cases occur in Africa [1]. In Ethiopia, about 68% (approximately 67.5 million people in 2015) of the population is at risk of getting malaria [2].

The transmission of malaria in Ethiopia is generally unstable and seasonal. There are two malaria transmission seasons in the country, one is the major transmission season that occurs between September and December, following the rain from June to August, and the second occurs between April and May, due to the February and March rains. Some localities may also experience perennial malaria transmission as the environmental and climatic situations permit the continual breeding of vectors in permanent breeding sites [2, 3].

In Ethiopia, there are four species of *Anopheles* mosquitoes which transmit malaria, namely, *Anopheles arabiensis*, *An. pharoensis*, *An. funestus* and *An. nili*. The former is the major vector, whereas the rest are secondary vectors [2].

The control of malaria involves education, vector control and chemotherapy, however, vector control has been recognized as the most effective [4]. To implement effective and locally suitable vector control measures, a detailed understanding on the ecology and behaviour of the local vectors and local malaria transmission dynamics is necessary [5]. Although malaria is prevalent in Addis Zemen, Libo-Kemkem Woreda [6, 7], information on the species composition, breeding sites, distribution and densities of malaria vectors is lacking. Therefore, this study aimed to document the species composition, larval habitat characteristics and the role of a small stream in maintaining larvae during the dry months.

## Methods

### Study area

This study was conducted from October 2011 to February 2012 in Addis Zemen town in Libo-Kemkem District, found in the South Gondar Zone of the Amhara Regional State. The district is situated at 37°15′36"E, 11°54′36"N, at an average elevation of 2000 m above sea level. The area receives a unimodal rainfall of approximately 1300 mm per year, mostly between June and August. The mean annual temperature is 19.7 °C. The district is divided into 30 *kebeles*, the smallest administration. According to the 2007 census report of the Ethiopian Central Statistical Agency (ECSA), its total population was 196,813 of which 88.9% live in rural areas.

**Fig. 1:**
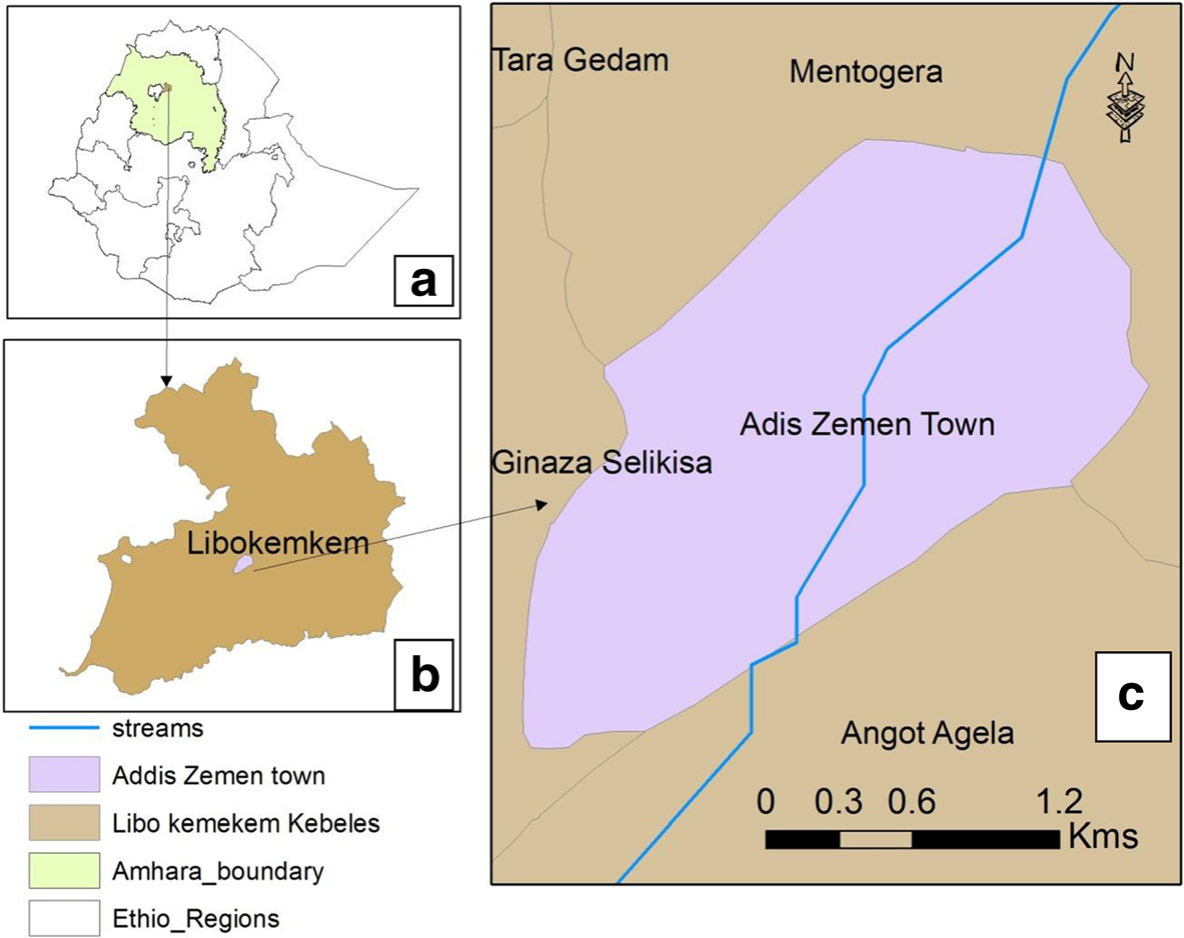
Location of the study area. **a** Ethiopia. **b** Libo-Kemkem. **c** Addis Zemen (Sheni stream is indicated in blue)

Addis Zemen is the capital town of Libo-Kemkem. It is divided into three *kebeles*, which are separated by road and Sheni stream (Fig. 1). Various government institutions and residential houses are located close to the stream. Local residents utilize Sheni stream for irrigation purposes, swimming, washing clothes and sand mining. The present entomological study covered all the three *kebeles* to understand the situation of malaria transmission. The study included inspecting Sheni stream if it maintains the aquatic stages of the malaria vector(s) in the dry months.

### Entomological studies

#### Larval sampling and species identification

Anopheline larvae were sampled twice monthly between October 2011 and February 2012, giving more focus on larval habitats along Sheni stream. Larvae were also sampled from habitats found out of Sheni stream in Addis Zemen. A standard larval dipper (11.5 cm diameter and 350 ml capacity), pipettes and a plastic tray were used in larva sampling. After inspecting for the presence of anopheline mosquito larvae, ten dips were taken from each mosquito breeding habitat [4, 8]. The water was left to settle for about 2 min after each subsequent dipping. Larvae of anophelines were separately taken from culicines and recorded according to their larval instar stages as first-, second-, third- and fourth-instar on prepared data sheets. Sampling was done by the same person in the morning (09:00–12:00 h) or afternoon (14:00–17:00 h) for about 60 min or less at each larval habitat throughout the sampling period. From the collected larvae, all third- and fourth-instars of anopheline larvae were killed and preserved in small vials containing 3% formalin solution.

Each larva was mounted on a glass slide separately in a drop of Gum-Chloral mounting medium and covered with a coverslip [9]. Identification of larvae was carried out using a compound microscope based on the key of Gillies & Coetzee [10].

#### Larval habitat characterization

For each habitat, environmental factors that could potentially be associated with the abundance of anopheline larvae were measured and recorded simultaneously with larval sampling. These characteristics included habitat depth, width and length, water pH, water temperature, exposure to sunlight, turbidity, vegetation type, water current, substrate type, whether the habitat is natural or man-made, presence of green algae, permanence of the habitat and distance of habitat to the nearest house.

Water temperature was measured using ordinary mercury thermometer and pH was measured using pH meter. A metal ruler was used to measure breeding habitat length, width and depth. The depth of each habitat was measured at three different points and the averages of these measures were recorded. Water current was determined by visual inspection and categorized as slow flowing or stagnant. Turbidity was observed by taking water in glass test tubes and holding against a white background to categorize as either clear or turbid. Exposure to sunlight was visually categorized as light and shade. The type and presence of aquatic vegetation was observed and recorded as emergent, floating, emergent plus floating and none if there was no vegetation at all. Type of substrate was observed and recorded as muddy, stone and soil, stone and sand gravel with little soil and stone. Distance to the nearest house was measured with a measuring tape when it is shorter than 100 m and by footsteps when it exceeded 100 m. These were then categorized into three classes (1, 0–100 m; 2, 100–300 m; and 3 for distances > 300 m) [11, 12].

The larval habitats were finally grouped according to their stability into temporary, semi-permanent and permanent habitats. Temporary are habitats that hold water for a short period of time (i.e. until approximately two weeks after larvae were collected from that habitat) whereas semi-permanent habitats are habitats that stay two to eight weeks by maintaining water. Permanent habitats; conversely, hold water for a longer period of time (i.e. for more than two months until the end of the sampling period) [12].

### Collection of indoor adult anophelines

Indoor anophelines were sampled using Center for Disease Control (CDC) light traps and pyrethrum spray sheet collections (PSC). The households were selected based on their distance from Sheni stream, which were 50–300 m from the stream. The houses and day of collection for PSC and CDC light trap collections were different. CDC light trap (John W. Hock Company, Gainesville, Florida, USA) collections were carried out twice monthly from six selected houses. For sampling of night-biting mosquitoes, light traps were hung on near the feet of sleeping persons and operated the whole night from dusk to dawn. Indoor resting mosquitoes were collected twice monthly using the pyrethrum spray sheet collection (PSC) method from five selected houses close to Sheni stream between 6:00–8:00 h [8].

Collected female *Anopheles* were classified according to their abdominal stages and also identified using the key in Gilles & Coetzee [10]. Specimens were preserved individually in Eppendorf tubes which contained silica gel and then transported to Aklilu Lemma Institute of Pathobiology (ALIPB) for further laboratory analysis.

### Collection of outdoor resting mosquitoes

Two artificial pit shelters [13] were constructed in a shaded site under a tree or large bush near to human dwellings near Sheni stream. In addition to pit shelters, a total of six clay pots (two per each *kebele*) were used to sample outdoor resting anopheline mosquitoes. Pots were placed in shaded places, such as under trees [14]. The outdoor traps were set apart from each other by at least 1 km. Resting mosquitoes, in these shelters, were collected twice a month with aspirator, torch and mosquito cage [4]. The time of collection for outdoor resting mosquitoes was from 1:00–3:00 pm. Mosquitoes were identified, categorized according to their abdominal status and preserved individually in Eppendorf tubes containing silica gel inside and transported to ALIPB.

### Identification of blood meal origins and circumsporozoite proteins of *Plasmodium* in anophelines

Blood meals of *Anopheles* captured from various collection methods were tested using enzyme-linked immunosorbent assay (ELISA) following the procedure of Beier et al. [15] at the Entomology Laboratory of ALIPB. The abdomen of freshly-fed *Anopheles* was ground in 50 μl PBS (0.01 M phosphate buffered saline), pH 7.4. Samples were then diluted in PBS (1:50) and 50 μl of the triturate added to each well of plates, which were then covered and incubated at room temperature for 3 h. At the same time, positive controls (human and bovine whole blood) and a negative control (prepared from laboratory reared unfed female *An. arabiensis*) were also added to specific wells. Each well was then washed twice with PBS containing 0.5% Tween 20 (PBS-Tw 20). This was followed by the addition of 50 μl host-specific conjugate [anti-host IgG conjugated in either peroxidase or phosphatase human IgG 1:2000, bovine 1:250 dilution in 0.5% boiled casein containing 0.025% Tween 20 (peroxidase conjugate for human, phosphatase conjugate for bovine)]. After 1 h, wells were washed three times with PBS-Tween 20. Finally, the absorbance at 414 nm was determined with microplate reader 30 min after the addition of 100 μl of ABTS peroxidase substrate. Each blood-meal sample was considered positive if the absorbance value exceeded the mean plus three standard deviations of the mean of three negative controls and also by observing color change (green color).

Similarly, *Anopheles* mosquitoes were tested for sporozoite infection using ELISA as described in Wirtzet al. [16]. The head and thorax of each of the *Anopheles* collected was ground in 50 μl of blocking buffer (BB) (IG-630). After grinding, each pestle was rinsed with two 100 μl BB to bring the total of titrate to 250 μl. Each well of a 96-well plate was coated with 50 μl of monoclonal *P. falciparum*, and *P. vivax*-210 and 247 capture-antibodies, which were then covered and incubated overnight at room temperature. Separate plates were used for each parasite species. The next morning the contents of the plates were aspirated, each well filled with blocking buffer and incubated at room temperature for 1 h. Then, the blocking buffer was aspirated and 50 μl of the mosquito triturate was added to the appropriate dried wells. Fifty microliters of positive (commercially prepared controls for each of the parasite) and negative (prepared from laboratory reared uninfected female *An. arabiensis*) controls were also added to specific wells at this time. After 2 h of incubation, the mosquito triturate was aspirated and the wells washed twice with PBS containing Tween-20. Then, 50 μl of monoclonal antibody peroxidase conjugate was added to each well and then after 1 h the plate washed three times with PBS-Tw. Finally, the absorbance was read at 405 nm using microplate reader 30 min after addition of ABTS substrate. Results were recorded as negative because the absorbance value did not exceed the mean plus three standard deviations of the mean of three negative controls, or also by observing no color changes.

### Data analysis

Data were entered in to Microsoft Excel 2003 and copied to data editor window of STATA version 11 software. The distribution of data for normality was checked by plotting a histogram. To compare mean larval density among habitat types and habitat characteristics with 3+ categories were analyzed using one-way analysis of variance (ANOVA). The mean density of each anopheline species among habitat characteristics with two categories was also compared by Student’s t-test for independent samples. The effect of environmental variables on the presence or absence of *Anopheles* larvae species in a given habitat was investigated using logistic regression after recording all the variables for each individual larva. Larval densities of a particular anopheline species from each breeding habitat were expressed as the number of larvae per 10 dips [4]. Linear regression was used to determine the predictor habitat characteristics associated with relative larval abundance of anopheline species. Significant associations observed during linear regression were further examined using multiple regression analysis. Data of adult anopheline species were analyzed using standard descriptive techniques. The mean daily density of anophelines collected from CDC traps was taken as the number of adult *Anopheles* collected**/**number of traps**/**number of nights.

## Results

### *Anopheles* larval collections

#### Anopheline species composition and habitat diversity

**Fig. 2:**
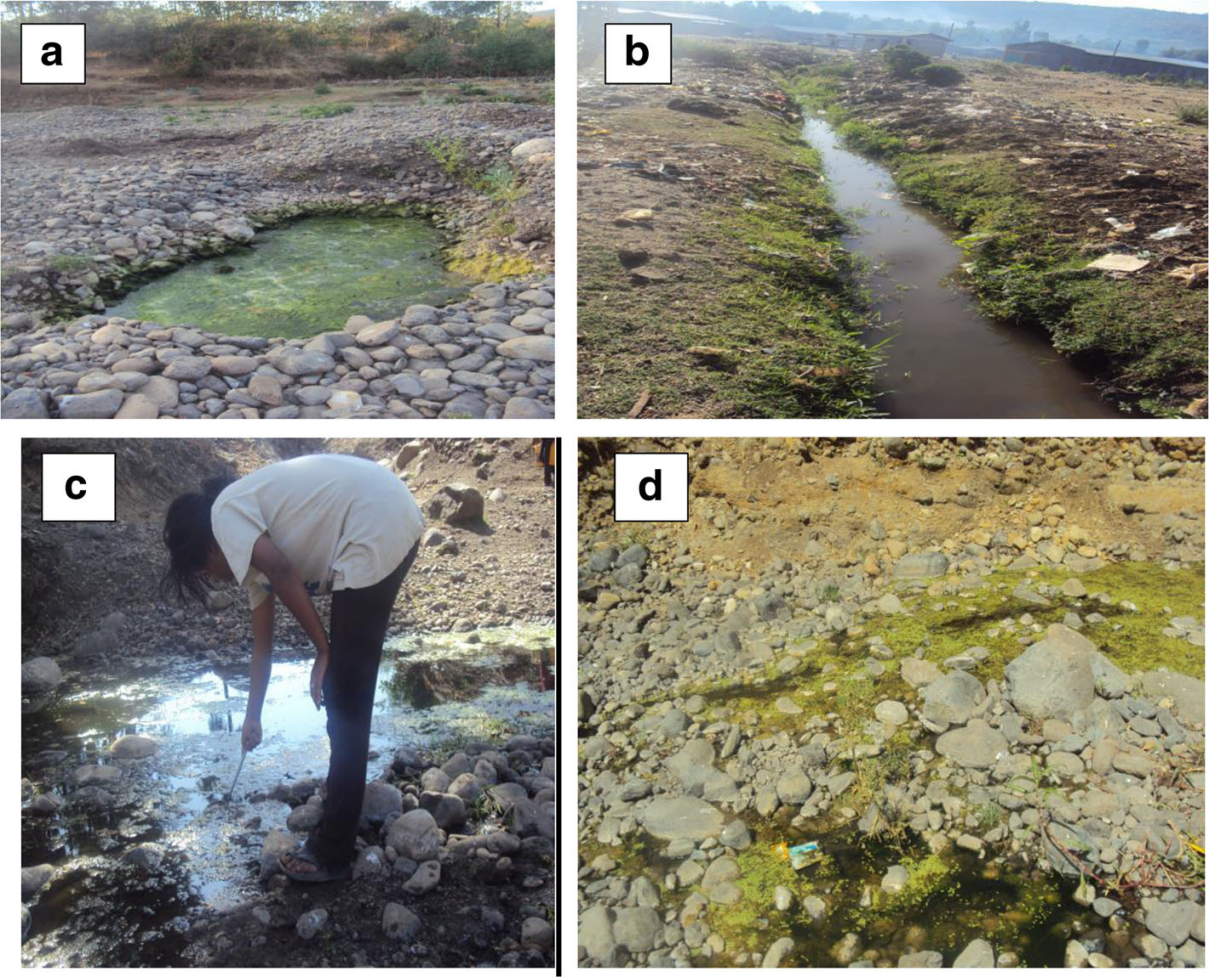
Image of different larvae habitats sampled during the study period (October 2011–February 2012). **a** Sand-mining pool in Sheni stream. **b** Drainage canal. **c**, **d** Natural pools in Sheni stream

A total of 6258 *Anopheles* larvae were collected from different breeding habitats, of which 3926 (62.7%) were early instars and the rest 2332 (37.3%) were late instars. Five species, namely *An. gambiae* (*s.l.*), *An. cinereus*, *An. demeilloni*, *An. christi* and *An. pretoriensis*, were identified (Table 1). The identity of *An. gambiae* (*s.l.*) is inferred from a study conducted in Gorgora which lies in the same geographical area with Addis Zemen. The most abundant species was *An. cinereus*, followed by *An. gambiae* (*s.l.*)*.* The proportion of the rest *Anopheles* was small. A total of 73 aquatic habitats were sampled, with most of them were from different sites of Sheni stream (Table 1). The habitats were sand mining and naturally created sand pools along Sheni stream (*n* = 66), rain pool (*n* = 4), hoof print (*n* = 1), drainage (*n* = 1) and irrigation canal (*n* = 1) (Fig. 2). The habitat on Sheni stream persisted throughout the study period, while the rest dried out in December. Larvae of the two predominant species, *An. gambiae* (*s.l.*) and *An. cinereus*, were collected most abundantly from Sheni stream and the stream was productive throughout the study period for both species. Sand mining pools of Sheni stream and drainage canal were inhabited by larvae of *An. demeilloni* and *An. christi*; however, they were scarce and absent from other types of habitats. *Anopheles pretoriensis* was very scarce and found only in sand mining pools of Sheni stream, together with *An. gambiae* (*s.l.*) and *An. cinereus*.

**Table 1:** Third- and fourth-instar *Anopheles* spp. mosquitoes collected from various breeding habitats in Addis Zemen (October 2011 - February 2012)

Habitat	No. of habitats analysed	*An. gambiae* (*s.l.*)	*An. cinereus*	*An. demeilloni*	*An. christi*	*An. pretoreinsis*	Total *n* (%)
Sheni stream							
Sand mining pool	2	272	881	14	3	1	1153 (49.4)
Natural pool	35	390	472	9	0	0	862 (38.0)
Spring	29	0	133	0	0	0	133 (5.8)
Rain pool	4	98	8	3	0	0	109 (4.7)
Hoof print	1	21	0	0	0	0	21 (0.9)
Drainage canal	1	2	0	2	3	0	7 (0.3)
Irrigation canal	1	0	20	0	0	0	20 (0.9)
Total	73	783	1514	28	6	1	2332 (100)

#### Mean larval density among habitat characteristics and habitat types

Variation in mean density of anopheline larvae among different categorical environmental characteristics is summarized in Table 2. The mean larva densities of *An. gambiae* (*s.l.*) were significantly higher from breeding habitats that were muddy (*F*_(3,69)_ = 2.85, *P* = 0.044), still (*t*_(71)_ = 1.55, *P* = 0.035) and temporary habitats (*F*_(2,70)_ = 23.19, *P* = 0.001) without mats of algae (*t*_(71)_ = 3.44, *P* = 0.001). Conversely, the mean density of *An. cinereus* was greater in slow flowing (*t*_(71)_ = 1.4, *P* = 0.005) and permanent habitats (*F*_(2,70)_ = 9.7, *P* = 0.0002) which had mats of algae (*t*_(71)_ = 8.39, *P* <  0.0001).

**Table 2:** Characteristics of larval habitats and mean densities per ten dips of anopheline larvae

Habitat characteristics	No. of habitats analysed	*An. gambiae* (*s.l.*) Mean ± SD (95% CI)	*An. cinereus* Mean ± SD (95% CI)
Permanence			
Permanent	23	5.5 ± 2.5	32.3 ± 5.5
Semi-permanent	21	0.7 ± 0 .4	30.5 ± 4.5
Temporary	29	22.2 ± 3.2	4.5 ± 1.3
Turbidity			
Clear	71	10.5 ± 1.9	21.3 ± 2.7
Turbid	2	19.5 ± 4.5	0
Distance from house			
0–100 m	47	10.9 ± 2.5	24.6 ± 3.7
100–300 m	15	11.4 ± 3.3	9.9 ± 3.4
≥ 300 m	11	9.1 ± 4.7	19.2 ± 5.5
Origin of habitat			
Natural	38	13.4 ± 2.8	16.8 ± 3.9
Man made	35	7.8 ± 2.3	25.0 ± 3.7
Presence of aquatic vegetation			
None	54	10.5 ± 2.3	21.7 ± 3.2
Emergent	7	9.1 ± 5.2	20.3 ± 9.6
Floating	9	16.2 ± 4.5	8.6 ± 3.9
Emergent and floating	3	2.0 ± 2.0	41 ± 10.2
Presence of algae			
Present	60	6.9 ± 1.7	25.2 ± 3.0
Absent	13	28.5 ± 4.1	0.3 ± 0.2
Type of substrate			
Muddy	5	29.2 ± 9.2	0
Sand and stone gravel with little soil	63	9.2 ± 1.9	23.1 ± 3.0
Stone and soil	3	7.0 ± 7.0	9.3 ± 5.6
Stone	2	18.0 ± 17.0	16.5 ± 11.5
Water current			
Slow flowing	25	5.3 ± 2.3	31.1 ± 15.4
Still	48	13.5 ± 2.5	15.4 ± 2.8

*Abbreviation*: *SD* standard deviation

The relative abundance of different species of anopheline larvae in different habitats was variable. ANOVA revealed the mean density of *An. gambiae* (*s.l.*) (*F*_(7,65)_ = 2.05, *P* = 0.045) to be significantly higher in rain pools and Sheni stream, whereas density of *An. cinereus* (*F*_(7,65)_ = 1.36, *P* = 0.039) was greater in the latter habitat.

#### Habitat characteristics associated with larval occurrence

All the habitats identified were found to be exposed to sunlight. *Anopheles gambiae* (*s.l.*) prefers to occur in temporary habitats compared to permanent habitats (OR = 27.80, 95% CI: 1.67–463.25) and muddy substrate types, rather than combination of soil and stone substrates (OR = 1.0135, 95% CI: 1.000–1.700). *Anopheles cinereus* was usually absent from habitats without mats of algae (OR = 0.016, 95% CI: 0.001–0.133).

#### Habitat characteristics associated with larval density

In linear regression analysis, the crude effect of each of the key environmental factors on larval density of anopheline was analyzed (Table 3). The relative larval density of anopheline larvae was negatively associated with changes in water temperature (16–34 °C) and pH (7–10). The abundance of *An. cinereus* larvae were also negatively associated with the water temperature and pH but positively associated with change in habitat length. The relative larval density of *An. gambiae* (*s.l.*) was negatively associated with change in habitat width. From eight categorical environmental factors analyzed, two were significantly and positively associated with total anopheline larval density. The relative abundance of both *An. gambiae* (*s.l.*) and *An. cinereus* larvae were significantly associated with four of the environmental variables: habitat water permanence, presence of algae, water turbidity and water current.

**Table 3:** Crude and adjusted effect of habitat characteristics using linear and multiple regression on the relative abundance of larval Anophelinae

Habitat characteristics	Linear regression			Multiple regression		
	Regression coefficient	SE	*P*-value	Regression coefficient	SE	*P*-value
*An. gambiae* (*s.l.*)						
Habitat width	15.66	2.07	0.034	-1.48	2.76	0.595
Permanence						
Semi-permanent	0.67	2.81	0.813			
Permanent	4.81	3.88	0.219	-17.83	8.00	0.030
Temporary	21.51	3.68	< 0.001	14.41	4.60	0.003
Presence of algae						
Present	6.87	1.76	< 0.001			
Absent	21.67	4.16	< 0.001	18.74	5.74	0.002
Water current						
Slow flowing	5.32	3.10	0.090			
Still	8.22	3.82	0.035	14.63	7.85	0.068
Turbidity						
Turbid	1.7	11.25	0.087			
Clear	17.7	11.41	0.432	27.29	12.83	0.038
Distance from house						
100–300 m	11.4	4.15	0.008			
0–100 m	-0.51	4.77	0.916	9.22	4.53	0.047
> 300 m	-2.31	6.38	0.719	4.66	5.39	0.392
*An. cinereus*						
Temperature	-1.73	0.58	0.004	-1.11	0.62	0.079
Habitat length	2.55	1.14	0.029	2.29	1.66	0.173
pH	-9.46	3.08	0.003	-6.46	3.86	0.100
Permanence						
Permanent	32.35	3.97	< 0.001			
Semi-permanent	-1.82	5.75	0.752	-0.17	12.34	0.989
Temporary	-27.90	5.32	< 0.001	-19.27	14.02	0.175
Presence of algae						
Present	25.17	2.72	< 0.001			
Absent	-24.86	6.45	< 0.001	-7.95	8.65	0.373
Water current						
Slow flowing	31.08	4.38	< 0.001			
Still	-15.73	5.41	0.005	-0.59	12.10	0.961
Habitat width	3.25	3.07	0.294	-9.08	4.26	0.038
Total anophelines						
Temperature	-1.07	0.53	0.047	-1.18	0.65	0.072
pH	-6.96	2.76	< 0.001	-2.90	4.01	0.473
Distance from house						
0–100 m	35.81	2.88	< 0.001			
100–300 m	-13.81	5.85	0.021	-11.31	7.27	0.126
> 300 m	-6.81	6.60	0.306	-7.6	7.47	0.311
Permanence						
Permanent	38.22	4.17	< 0.001			
Semi-permanent	-6.88	6.03	0.258	-19.08	12.85	0.143
Temporary	-10.80	5.58	0.057	-23.02	14.59	0.121
Habitat width	-1.25	2.72	0.647	-10.66	4.43	0.020

*Abbreviation*: *SE* standard error

Further, in multiple regression analysis after adjustment for environmental characteristics, the relative abundance of total anopheline larvae was negatively associated with change in habitat width, which was also true for *An. cinereus* larval abundance. The abundance of *An. gambiae* (*s.l.*) larvae was positively associated with clear and temporary habitats that had no mats of algae and located between 0 and 100 m from human dwellings, but negatively associated with permanent habitats.

### Adult collections

A total of 182 adult female anopheline mosquitoes were captured using various methods of collections (Table 4). Three *Anopheles* species, namely *An. gambiae* (*s.l.*), *An. cinereus* and *An. demeilloni*, were identified. Unlike larval sampling, *An. gambiae* (*s.l.*) was the most abundant species followed by equal number of *An. demeilloni* and *An. cinereus*. The two other species that were scarcely obtained in larval collection were absent in adult collections.

**Table 4:** Adult *Anopheles* mosquitoes collected by different methods in Addis Zemen (October 2011–February 2012)

Method of collection	*An. gambiae* (*s.l.*)	*An. cinereus*	*An. demeilloni*	Total *n* (%)
CDC traps	79	35	39	153 (84.1)
PSC	6	2	0	8 (4.4)
Pit shelter	3	9	8	20 (11.0)
Clay pot	0	1	0	1 (0.5)
Total *n* (%)	88 (48.4)	47 (25.8)	47 (25.8)	182 (100)

### Indoor collections

**Fig. 3:**
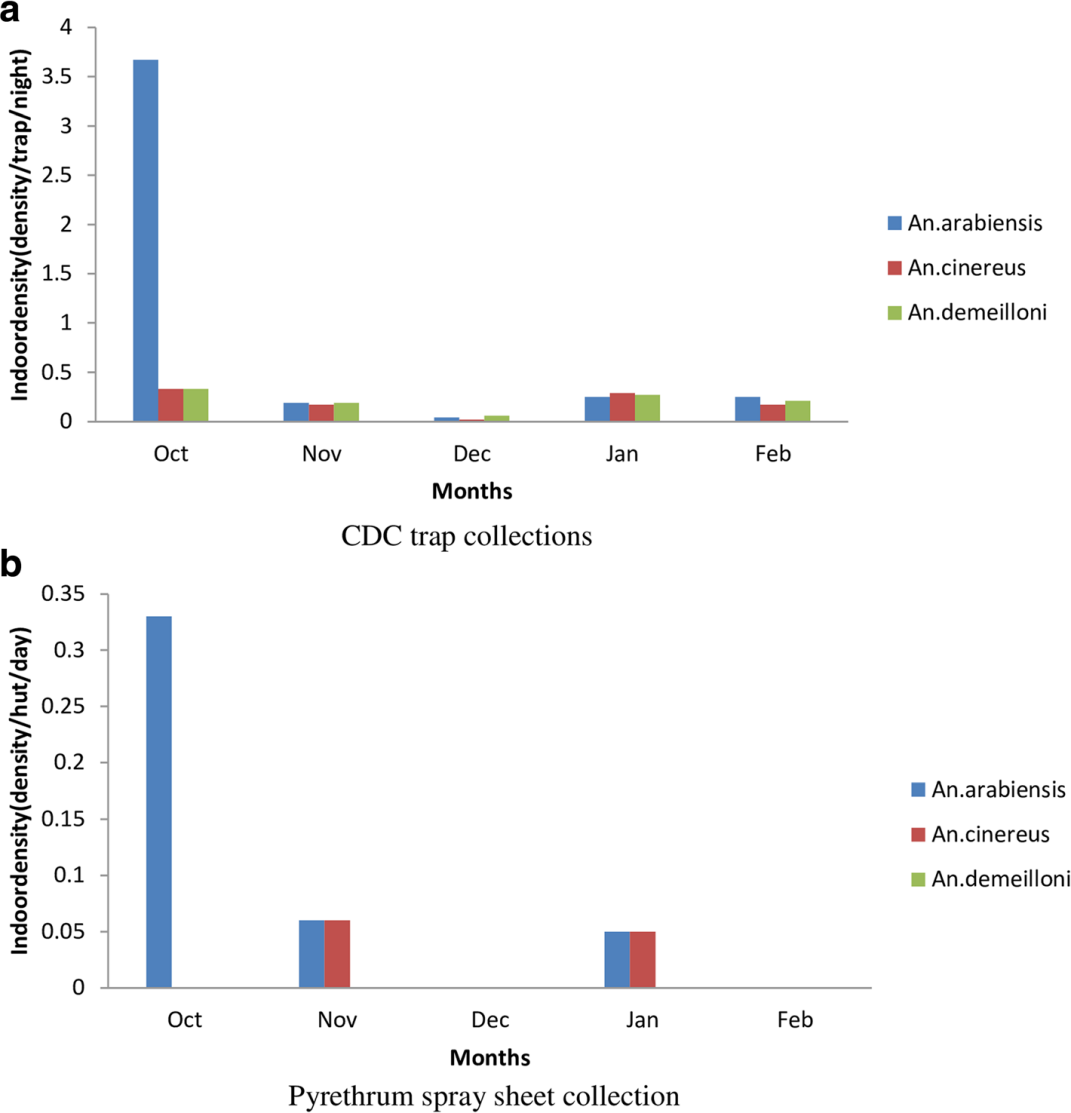
Temporal distribution of anophelines indoor density based on two methods (October 2011 - February 2012)

A total of 161 *Anopheles* representing three species [*An. gambiae* (*s.l.*), *An. cinereus* and *An. demeilloni*] were caught using CDC light traps and pyrethrum spray sheet collection (Table 4). Very low numbers of *An. gambiae* (*s.l.*) and *An. cinereus* were obtained from 44 houses inspected using PSC method. *Anopheles gambiae* (*s.l.*) was more predominant in human dwellings than *An. cinereus* and *An. demeilloni*. The mean daily density in CDC light traps (number of anopeline species/trap/night) was 0.87 for *An. gambiae* (*s.l.*), 0.19 for *An. cinereus* and 0.21 for *An. demeilloni*. The density of *An. gambiae* (*s.l.*) varied between the months; the highest density was in October whereas the lowest was in December (Fig. 3a, b).

### Outdoor collections

Attempts to collect *Anopheles* from 17 visits of 2 pit shelters and 32 visits of 6 clay pots resulted in capturing nine *An. cinereus*, eight *An. demeilloni* and three *An. gambiae* (*s.l.*)*. Anopheles gambiae* (*s.l.*) was collected only in February from pit shelters. Clay pots were not productive, with only one *An. cinereus* collected throughout the sampling period.

### Blood meal source and test for circum-sporozoite proteins

Blood meals of 29 freshly fed anophelines, of which the majority was sampled from indoor locations, were tested to determine their source of blood meals. The small number of blood meals of *An. gambiae* (*s.l.*) indicated the source as both human and bovine (Table 5). The other two species also appear to feed on both human and cattle, however, the blood meals were predominantly taken from bovine. A total 182 anophelines captured by four different methods, 88 *An. gambiae* (*s.l.*), 47 *An. cinereus* and 47 *An. demeilloni*, were tested to detect circum-sporozoite proteins of *Plasmodium falciparum* and *P. vivax* by ELISA method, however, none were positive.

**Table 5:** Blood meal sources of *Anopheles* from Addis Zemen (October 2011 - February 2012)

Species	No. tested	Blood meal source			
		Human *n* (%)	Bovine *n* (%)	Mixed *n* (%)	Unidentified *n* (%)
*An. gambiae* (*s.l.*)	7	2 (28.6)	2 (28.6)^a^	0	3 (42.8)
*An. cinereus*	10	1 (10)	9 (90)^a^	0	0
*An. demeilloni*	12	0	10 (83.4)^b^	1 (8.3)	1 (8.3)
Total	29 (100)	3 (10.3)	21 (72.5)	1 (3.4)	4 (13.8)

^a^One was from outdoor collections

^b^Two were from outdoor collections and the rest were from indoor collections

## Discussion

This study provided baseline information for the species composition of anophelines, types of larval breeding habitats and adjoining characteristics as well as some entomological indicators in Addis Zemen in relation to malaria transmission. The presence of *An. gambiae* (*s.l.*) (presumably *An. arabiensis*), the principal vector of malaria in the country [2], and four other non-vectors (*An. cinereus*, *An. demeilloni*, *An. christi*and and *An. pretoriensis*) is ascertained from both larval and adult sampling. Larvae of *An. gambiae* (*s.l.*) were the second most abundant in almost all habitats, including sun-lit pools formed at the bed and edges of Sheni stream, rain pools, hoof prints and drainage canals. All the types of habitats reported here have been previously documented in Ethiopia as well as elsewhere in Africa [11, 17–19]. Sheni stream is the most common breeding site in the area and the density of *An. gambiae* (*s.l.*) and *An. cinereus* was higher here than other breeding sites. This is inconsistent with previous study in Eretria [18, 19]. Sand mining and naturally created pools along Sheni stream captured *An. gambiae* (*s.l.*) as the water is clear and sunlit. This observation is similar to the findings noted by Keneaet al. [11] in Ziway area. Similarly, higher density of this species was also sampled from rain pool habitats.

Like *An. gambiae* (*s.l.*), *An. cinereus* abundantly breeds in Sheni stream, while the contrary was noted for *An. demeilloni*, *An. christi* and *An. pretoriensis.* All these species are regarded as highland mosquitoes, except for *An. gambiae* (*s.l.*) and *An. pretoriensis* whose distribution extends to the lowlands [20, 21].

Multiple regression analysis revealed that clear and sun-lit temporary habitats are positively associated with the abundance of *An. gambiae* (*s.l.*). This agrees with recent findings in Ethiopia which indicated that *An. arabiensis* breeds in clear, temporary and often sun-lit pools of water [11, 21]. This could be due to inert particles suspended in the larval environment, which may prevent larval mosquitoes from feeding, being found less in clear water than in turbid water [22–24]. In contrast, other studies showed larval density of this species to be positively associated with turbid semi-permanent habitats [24, 25]. The positive association of *An. gambiae* (*s.l.*) larval density with habitats devoid of mats of green algae reported here may be due to the exposure of habitats with muddy substrate to sunlight provide favorable conditions for the survival of bacteria from which larvae get their nutrients [26, 27]. The negative association of permanent habitats with the abundance of *An. gambiae* (*s.l.*) is similar to the findings of Keneaet al. [11]. This may be because of larval predation is more prevalent in large, permanent habitats [22–24]. *Anopheles gambiae* (*s.l.*) is positively associated with habitats located between 0 and 100 m from human dwellings, which is one of the strong predictors for indoor *Anopheles* abundance [28]. Change in habitat width, however, is negatively associated with the abundance of *An. cinereus*.

Although the number is small, adult *Anopheles* collections contained more *An. gambiae* (*s.l.*) than the other species, the majority of which were captured from CDC light traps indoors indicating possible host-seeking behaviour, although they may have been attracted by light from the trap. This might be consistent with its anthropophilic and endophagic behaviorur noted by other investigators [21, 29]. The mean daily density in CDC light traps of this species was relatively high compared to the study conducted in Fuchucha & Jarso [30], which was 0.3/trap/night. Even though the number of fresh fed *An. gambiae* (*s.l.*) tested for blood meal analysis was very low, the few positive reactions exhibited both the zoophilic and anthropophilic behaviour of the vector, which is typical of its biting behaviour [10] and is similar to a number of studies in Ethiopia [21, 30, 31]. All these indicated that this species might be responsible for local malaria transmission in the study area. The outdoor density of this species in Addis Zemen could not be determined, as only three mosquitoes were collected from pit shelters. The absence of sporozoite infection could also be due to the very small number adult mosquitoes tested.

*Anopheles cinereus* and *An. demeilloni* were the second greatest in adult sampling, after to *An. gambiae* (*s.l.*), and the majority of these tested for blood meal analysis showed a preference towards cattle feeding showing their zoophilic and poor anthropophilic behaviorur. This is in agreement with the study conducted in highlands of western Kenya [32]. These species are also widely distributed in east African highlands, at altitudes ranging from 1400 to 2500 m [10].

Only one *An. cinereus* was caught resting in clay pots in outdoor collection, showing very little attraction to man-made habitats. However, in western Kenya, more mosquitoes were captured from clay pots (37%) than pit shelters [14].

Adults of *An. gambiae* (*s.l.*), *An. cinereus* and *An. demeilloni* were present in collections throughout the study period. Moreover, larvae of the former two species continued to survive in Sheni stream during the dry months, showing the importance of this stream in providing suitable condition for the survival of the two species, particularly to *An. gambiae* (*s.l.*), during the period when other breeding sites perish. The presence of *An. gambiae* (*s.l.*) in both stages during the entire study period indicates that active transmission of malaria might take place throughout the entire year. Therefore, further study on the prevalence of malaria in conjunction with anophelines is required to better describe the disease.

## Conclusions

The present study demonstrated the preferred anopheline larval habitats and best predictor environmental factors for larval abundance. Sheni stream, present in the study area, plays an important role in maintaining *An. gambiae* (*s.l.*) and other *Anopheles* species. In addition, this study indicates that the presence of the principal malaria vector, *An. gambiae* (*s.l.*), in the country in the study area from both larval and adult collections. Since the collection of adult mosquitoes was low, owing to the brief period of study and the dry season, a more detailed and year-round investigation is required to gather appropriate and relevant entomological indices of transmission towards contributing knowledge-based strategy for effective vector control management.

## Abbreviations

ALIPB: Aklilu Lemma Institute of Pathobiology
CDC: Center for Diseases Control and Prevention
ELISA: Enzyme-linked immunosorbent assay
PBS: Phosphate buffered saline
PSC: Pyrethrum spray sheet collection
